# Gelatin-based biomaterials and gelatin as an additive for chronic wound repair

**DOI:** 10.3389/fphar.2024.1398939

**Published:** 2024-05-01

**Authors:** Hongwei Cao, Jingren Wang, Zhanying Hao, Danyang Zhao

**Affiliations:** ^1^ Department of Otorhinolaryngology, The First Affiliated Hospital of China Medical University, Shenyang, China; ^2^ Department of Prosthodontics, Affiliated Stomatological Hospital of China Medical University, Shenyang, China; ^3^ Department of General Surgery, The Fourth Affiliated Hospital of China Medical University, Shenyang, China; ^4^ Department of emergency Surgery, The Fourth Affiliated Hospital of China Medical University, Shenyang, China

**Keywords:** biomaterials, gelatin, wound repair, hemostasis, antibacterial, antiinflammatory

## Abstract

Disturbing or disrupting the regular healing process of a skin wound may result in its progression to a chronic state. Chronic wounds often lead to increased infection because of their long healing time, malnutrition, and insufficient oxygen flow, subsequently affecting wound progression. Gelatin—the main structure of natural collagen—is widely used in biomedical fields because of its low cost, wide availability, biocompatibility, and degradability. However, gelatin may exhibit diverse tailored physical properties and poor antibacterial activity. Research on gelatin-based biomaterials has identified the challenges of improving gelatin’s poor antibacterial properties and low mechanical properties. In chronic wounds, gelatin-based biomaterials can promote wound hemostasis, enhance peri-wound antibacterial and anti-inflammatory properties, and promote vascular and epithelial cell regeneration. In this article, we first introduce the natural process of wound healing. Second, we present the role of gelatin-based biomaterials and gelatin as an additive in wound healing. Finally, we present the future implications of gelatin-based biomaterials.

## 1 Introduction

The skin is the largest organ of the human body and the first barrier to protect the body from external interference (Hassanshahi et al., 2022). However, skin integrity is often compromised because of trauma, burns, and other factors, leading to a breakdown in the defense barrier. Muscles, organs, and other human body tissues interact directly with the harsh external environment, which not only facilitates infection around the wound but also induces pain and harms the tactile organs (Huang et al., 2022a). Wounds are mainly divided into acute and chronic wounds (Falanga et al., 2022). Although there is no clear boundary, a wound that does not pass regular repair within 3 months is generally accepted as a chronic wound (Izadi and Ganchi, 2005). Acute wounds usually heal within a few weeks and are caused by mechanical and thermal injuries (Joorabloo and Liu, 2023). Chronic wounds are mainly mediated by infection and necrotic tissue; their healing times vary from several months to permanent non-healing (Fallah et al., 2021). Chronic wound treatment not only imposes a severe financial burden on patients but also causes tremendous psychological pressure on patients (Hu et al., 2019). Approximately 2%–4% of annual medical expenses in developed countries are reportedly devoted to chronic wound treatment (Olsson et al., 2019). In the United States, approximately six million people suffer from chronic wounds each year. The number of patients suffering from chronic wound non-healing is still on the rise due to factors such as diabetes (Powers et al., 2016; Keni et al., 2023). Therefore, promoting chronic wound healing is one of the urgent issues that clinicians must investigate.

**SCHEME 1:**
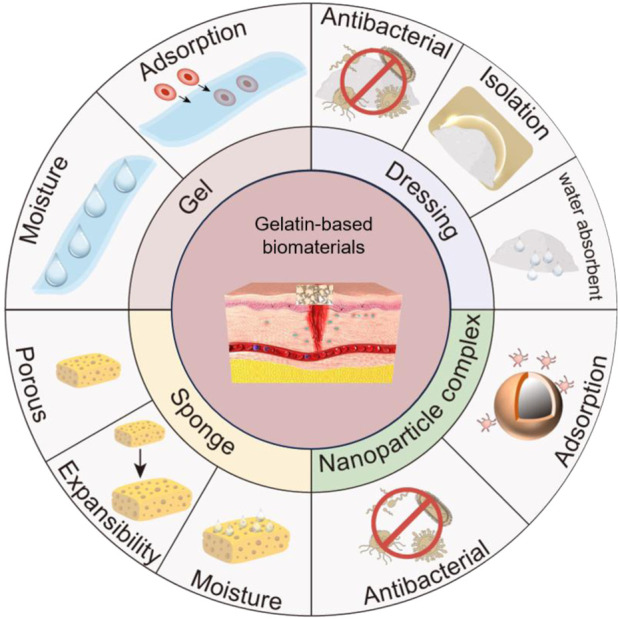
Gelatin-based biomaterials promote chronic wound healing. Gelatin-based nanocomposites enhance peri-wound antimicrobial properties and promote chronic wound repair. Gelatin-based sponges are porous, absorbent, and expandable, absorbing water around the wound to promote chronic wound healing. Gelatin-based hydrogels can adsorb red blood cells and platelets, initiate blood clotting, and promote wound healing. Gelatin-based dressings have antimicrobial properties, insulate bacteria from wounds, and promote chronic wound healing. The drawing software for Scheme 1 is Microsoft PowerPoint.

Chronic wounds are formed because of disturbances in the regular healing process, substantially prolonging the healing time. The Wound Healing Association divides chronic wounds into four categories based on etiological causes: pressure, venous, arterial, and diabetic ulcers (Kirsner, 2016). Chronic wounds are often accompanied by severe skin and tissue damage, resulting in poor resistance and local trophic impairment, thereby aggravating wound non-healing (Wilkinson and Hardman, 2020). Wound healing is predicated primarily on the extracellular matrix (ECM), which provides the wound its structure (Amaral et al., 2018). However, bacterial and inflammatory factors around chronic wounds do not offer adequate stability to support ECM exchange (Liu et al., 2023a). Wound healing is an important part of the biomedical field, but its development remains in its early stages (Nandhakumar et al., 2022). With the development of biomedicine, gelatin has been used in anti-tumor, tissue repair, regenerative medicine, and many other fields because of its biocompatibility and degradability (Xia et al., 2022a; Xia et al., 2022b; Xia et al., 2023). Gelatin—non-immunogenic and resembles ECM in structure—is essential in wound healing (Elangwe et al., 2023). In this review, we present the application of gelatin-based biomaterials and gelatin as an additive in wound healing (Table 1; Scheme 1) and analyze the challenges. Finally, we present the future applications of gelatin-based biomaterials.

**TABLE 1:** Gelatin-based biomaterials for chronic wound repair.

Function	Name	Main composition	Material properties	Results	Reference
Hemostasis	Gel adhesive	Sodium alginate and protocatechualdehyde	The gel adhesive had an energy storage modulus of 2400 Pa	Hydrogels have antioxidant and good hemostatic effect	Huang et al. (2022b)
	PCGS	Chitosan/gelatin, proanthocyanidins	The zeta potential was 5 ± 1.55 mV	PCGS achieved excellent hemostasis performance in rat femoral artery injury models	Yang et al. (2017)
	GTT-3 gelatin	Tannins/gelatin and glutamine transferase	GTT-3 hydrogels had an initial length of 20 mm, a tensile length of 70 mm, and a bond strength of up to 8.5 kPa	GTT-3 hydrogels have good biocompatibility, immediate adhesion, and hemostatic and therapeutic healing effects	Wang et al. (2019)
	HI/DA- gelatin	Dopamine, hyaluronic acid, gelatin	The adhesion strength of HI/DA-gel was 27 ± 3 kPa	HI/DA-Gel has high adhesion, therapeutic hemostatic, and wound-healing properties	Zhou et al. (2021)
	GT/Ag cryogel	Gelatin/silver nanoparticles	AgNPs had a particle size of 10–20 nm and a gelatin/silver nanoparticle compressive strength of 7 kPa	GT/Ag cryogel has excellent antimicrobial properties and effective absorption of wound exudates	Luo et al. (2022)
	GA/ODex/EPL2-B	Gelatin, methacryloyl, dextran oxide, polylysine	The tensile strength of a GA/ODex/EPL2-B group was 16.9 ± 0.44 kPa	GA/ODex/EPL2-B accelerates skin tissue epithelialization, collagen deposition, and angiogenesis by inhibiting bacterial growth	Tang et al. (2020)
Antibacterial	PCL-Cur/GEL-TH core-shell nanofiber membrane	Polycaprolactone, curcumin, gelatin, tetracycline hydrochloride	The PCL-Cur/GEL-TH core-shell nanofiber membrane had a diameter of 178 ± 25 nm	PCL-Cur/GEL-TH enables the continuous release of TH and Cur, maintaining anti-inflammatory and antioxidant effects	Rezaii et al. (2019)
	QCSMOF-Van	Quaternary ammonium salt chitosan, sodium alginate, vancomycin	QCSMOF-Van had a size of about 700 nm	QCSMOF-Van’s versatile strategy with rapid antibacterial, anti-inflammatory, nerve regenerative, and angiogenic abilities contributes to the rapid healing of chronic wounds	Gupta et al. (2016)
	G@Fe/PGD wound dressings	Ferro-metal organic framework, PCL/gelatin/glucose composite fiber web	QCSMOF-Van also had high adhesion strength (34.146 ± 5.032 kPa) and compressive strength (74.667 ± 9.504 kPa)	QCSMOF-Van can promote wound anti-infection and promote wound healing	Porfire et al. (2014)
	AgPOM nanoparticles	Polyoxometallic oxides (AgPOM), urea, gelatin, and tea polyphenols	AgPOM nanoparticles had a particle size of about 70 nm	The hydrogels have high bactericidal ability against drug-resistant *Staphylococcus aureus*, revealing a significant therapeutic effect on infected wounds by synergistic photothermal/chemokinetic therapy	Huang et al. (2022d)
	Gel&Fuc-TAHydrogel	Gelatin - fucoin - tannins	Gel&Fuc-TA obtained a porous structure (1.9–5.26 μm)	The Gel&Fuc-TA hydrogels controlled the release of Fuc and TA, providing good antioxidant and antibacterial properties	Zhang et al. (2023)
	Alg-DA gelatin bracket	Dopamine, alginate, gelatin	The compressive strength of the hydrogel stent was 1.42 ± 0.24 Mpa	The hydrogel scaffolds had potent antibacterial activity against both Gram-positive and Gram-negative bacteria	Lu et al. (2022a)
Anti-inflammatory	Inflammatory reactive hydrogel	3-Carboxyphenylboronic acid, polyvinyl acetate, nimesulide	The Inflammatory reactive hydrogels had a diameter of 64.25 nm and a potential value of 23.6 mV	Inflammatory reactive hydrogels promote the healing of infected wounds by sequential hemostasis and antibacterial and anti-inflammatory processes	Akita (2019)
	Tsg-THA&Fe Hydrogel	Fe3+ and 2,3,4-Trihydroxybenzyl, tilapia skin gelatin	The maximum compressive stress of Tsg-THA&Fe hydrogels was 23 N	Tsg-THA&Fe hydrogels reduced the expression of pro-inflammatory cytokines TNF-α, IL-2, IL-6, and IL-8β and upregulated the expression of IL-1, Arg-10, and TGF-β	Zheng et al. (2000)
	C60@PDA/GelMA Hydrogel	Gelatin methacryloyl, PDA	C60@PDA/GelMA hydrogels exhibited a low compressive strength of 3.7 kPa and a compressive fracture strain of 135%	Hydrogels improve healing by relieving oxidative stress and inflammation and promoting reepithelialization, collagen deposition, and neovascularization	Wang et al. (2021)
	Complex hydrogel	Curcumin-terminated silver nanoparticles, gelatin, chitosan, polydopamine	The complex hydrogels had a high adhesion strength of 55 ± 1 kPa	Complex hydrogels have bactericidal and anti-inflammatory properties	Zhang et al. (2018b)
	PH/GMs@bFGF&PDA	Basic fibroblast growth factor, polydopamine, gelatin, polyvinyl acetate (PVA), hyaluronic acid	The adhesion of PH/GMs@bFGF&PDA was 101.46 ± 10.88 kPa	PH/GMs@bFGF&PDA significantly relieves inflammation and promotes the secretion of collagen I, thereby enhancing wound healing through its synergistic effect of shape adaptability and high adhesion properties	Yu et al. (2016)
Promotes vascular regeneration	GelMA + cADSC-CM	Gelatin methacrylic, fat-derived mesenchymal stem cells	The pore size of the GelMA + cADSC-CM hydrogels was 342.3 μm	GelMA + cADSC-CM accelerates wound healing and angiogenesis of aged skin *in vivo*	Chen et al. (2012)
	ADA-GEL/BBG/ASX composite	alginate dialdehyde–gelatin, borate bioactive glass, astaxanthin	BBG particle sizes ranged from 10 to 100 μm	ADA-GEL/BBG/ASX composites are an attractive biomaterial for the development of multipurpose wound healing structures through 3D printing	Balasubramanian et al. (2017)
	DMN@TH/rh-EGF	Tetracycline hydrochloride, recombinant human epidermal growth factor, gelatin, carboxymethyl chitosan, hyaluronic acid	The height of DMN@TH/rh-EGF was ≈780 μm	DMN@TH/rh-EGF patch can suppress inflammation and promote angiogenesis, collagen deposition, and tissue regeneration during wound healing	Biswal et al. (2016)
	CvMN	Polyvinyl acetate, gelatin methacryloyl, *Chlorella vulgaris*	The CvMN height was 600 μm	CvMN has shown good therapeutic efficacy in treating skin wounds in a mouse model of diabetes	Liu et al. (2023b)
	PU/Gel/KTC mats	Hydrogen sulfide, polyurethane (PU) and gelatin	PU/Gel/KTC mats achieved water absorption of 400% of its volume	PU/Gel/KTC mats can accelerate granulation tissue formation, enhance collagen deposition, and promote angiogenesis	Akter (2016)
	Fibrous Membrane Scaffolds	PLGA/Gelatin/Hyaluronic Acid Fibrous	The average fiber diameter of PG was 1014 ± 15 nm	Fibrous Membrane Scaffolds treat wounds by modulating the immune response, promoting angiogenesis, and reducing scarring at the wound site	Hu et al. (2016)
Promotes epidermal regeneration	GEL/APS NFM	Gelatin and astragalus polysaccharides	The average diameter of GEL/APS NFM was 773 nm, and its elongation reached 514.6%	GEL/APS NFM can exert anti-inflammatory, procollagen deposition, and pro-angiogenic effects	Lasram et al. (2015)
	Gelatin microspheres	GelMA, umbilical cord mesenchymal stem cells	—	Gelatin microspheres can cause new collagen deposition and angiogenesis, accelerating wound healing and skin tissue regeneration	Liu et al. (2011)
	GelMA/Mg/Zn hydrogel	zinc and magnesium granules, GelMA	GelMA/Mg/Zn hydrogels had a pore size of 20–100 μm	GelMA/Mg/Zn hydrogels can promote wound reepithelialization and angiogenesis and promote wound healing	Li et al. (2022)
	G-S hydrogel	GelMA, Silk fibroin	G-S hydrogels had a porous structure with average pore sizes of 226.54 ± 41.86 μm	GelSilMA (G-S) hydrogels can help accelerate wound closure by improving the microenvironment, promoting epidermal tissue regeneration and endogenous collagen production	Amberg et al. (2018)

## 2 The wound healing process

**SCHEME 2:**
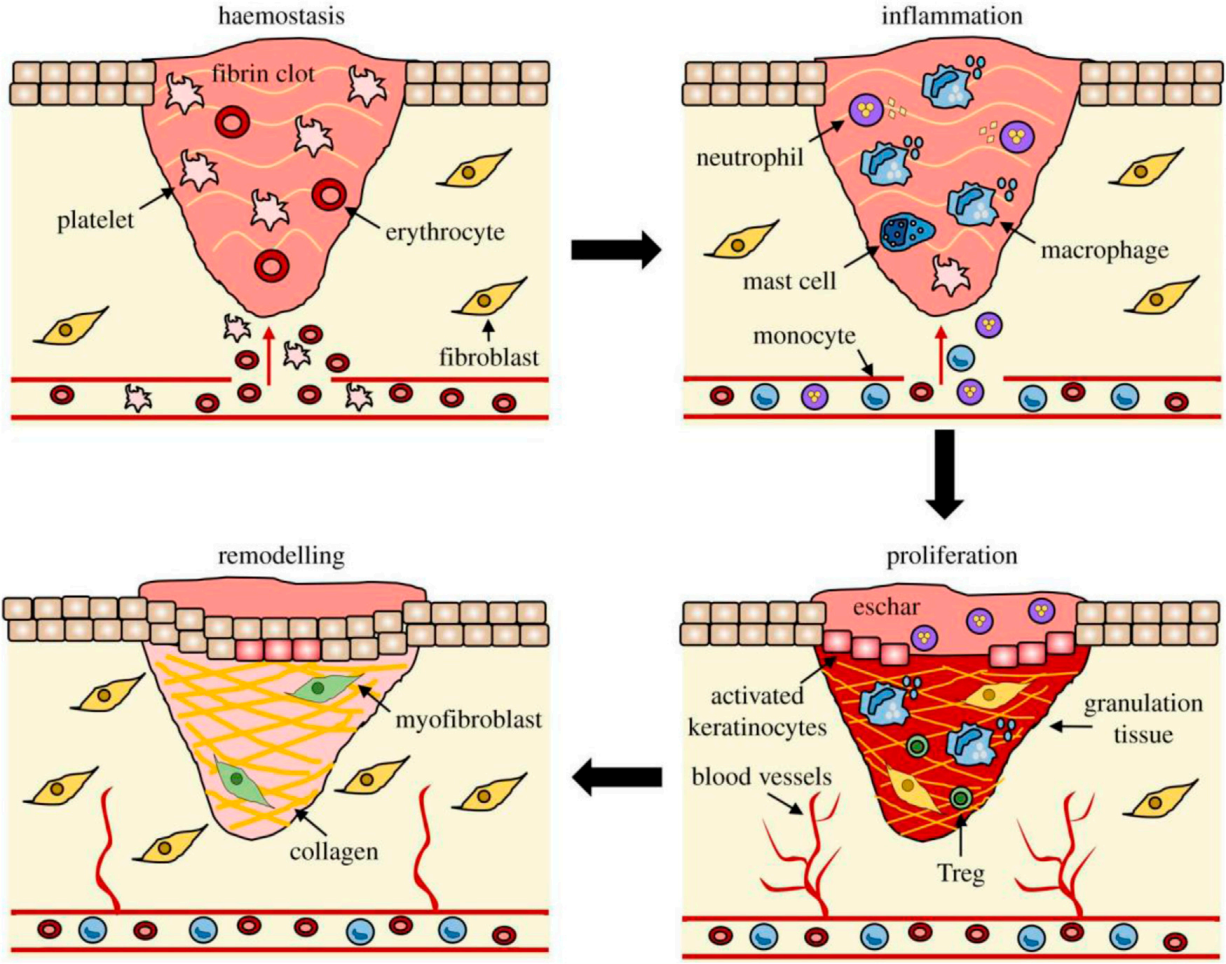
The process of wound healing. The stages of wound repair and their major cellular components. Wound repair begins with hemostasis, where a platelet plug prevents blood loss, forming a preliminary fibrin matrix. Inflammation then ensues to remove debris and prevent infection, commencing with neutrophil influx promoted by histamine release from mast cells. Monocytes arrive later and differentiate into tissue macrophages to clear remaining cell debris and neutrophils. During the proliferative phase, keratinocytes migrate to close the wound gap, blood vessels reform through angiogenesis, and fibroblasts replace the initial fibrin clot with granulation tissue. Macrophages and regulatory T cells (Tregs) are crucial for the proliferative phase. Finally, fibroblasts further remodel the deposited matrix, blood vessels regress, and myofibroblasts cause overall wound contraction. Reproduced with permission from (Wilkinson and Hardman, 2020).

Wound healing is a complex process that involves interactions between different cell types, cytokines, antioxidants, and ECMs (Scheme 2) (Krizanova et al., 2022). The initial step of wound healing is arteriole contraction, which reduces local blood flow (Huang et al., 2022a). Platelets release serotonin and prostaglandins, further promoting vasoconstriction and blood stagnation. Platelets are adsorbed by collagen fibers and aggregate into blood clots, forming thrombocytosis and initiating clotting (Wang et al., 2018a). Subsequently, there is an increase in vascular permeability, which mediates the migration of inflammatory cells to the site of injury by releasing inflammatory factors; thus, wound healing enters the inflammatory phase. Neutrophils are the first inflammatory cells to appear around the wound (Almadani et al., 2021). Inflammatory cells release inflammatory factors while engulfing necrotic material around the wound, causing the wound to swell, redness, warmth, and pain (Yusuf Aliyu and Adeleke, 2023). The inflammatory response is designed to build an immune barrier against bacteria entering the wound (Strodtbeck, 2001). With the dilation of blood vessels during the inflammatory phase, nutrients, antibodies, and growth factors enter the wound’s surroundings. With the proliferation of newly formed blood vessels and fibroblasts, wound healing enters a proliferative phase (Zhu et al., 2023). The proliferative phase is usually inseparable from the inflammatory phase, mainly the organic inflammation stage (Ridiandries et al., 2018). Epithelial regeneration, neovascularization, and granulation tissue formation are prerequisites for skin barrier rebuilding (Su et al., 2021). Signaling between fibroblasts and the microenvironment leads to ECM deposition to fill wounds (Patel et al., 2019). The remodeling phase is the final crucial step in wound healing, in which the ECM matures into scars and gains tensile strength (Sheir et al., 2022). During this phase, collagen fibers are degraded and reordered, capillaries are reduced and converged into large vessels, and granulation tissue organizes into scar tissue (Huang et al., 2022a).

## 3 Properties of gelatin

Gelatin is obtained through the acid, alkali, or hot water hydrolysis of natural collagen, and its main structure is similar to collagen (Tan et al., 2018). The conversion of collagen into gelatin produces molecules with varying molecular weights so that gelatin can be hydrolyzed into amino acids and peptides by most proteolytic enzymes (Mikhailov, 2023). In the process of collagen hydrolysis to form gelatin, a large number of functional groups will be leaked. These functional groups bind to crosslinkers or targeted ligands to impart different properties to gelatin (Wang et al., 2012). For this reason, gelatin is biodegradable and biocompatible (Lukin et al., 2022). Because of gelatin’s 1:1:1 ratio of anionic, cationic, and hydrophobic groups, its rheological properties and thermal stability are almost constant between pH 5 and 9 (Milano et al., 2023). Approximately 13% of gelatin’s polypeptide chain consists of positively charged amino acid residues, 12% negatively charged amino acid residues, and 11% hydrophobic residues (Elzoghby, 2013). Because of these residues, gelatin often exhibits amphoteric behavior, demonstrating different physicochemical properties at different pH values (Stagnoli et al., 2023). The amphoteric behavior characteristics of gelatin have been developed to deliver various drugs to treat cancer (Aljabali et al., 2022). Another characteristic of gelatin is viscosity, which increases with polymer concentration and decreases with temperature and pH (Milano et al., 2023). Gelatin is also a heat-sensitive material, often used as a sol-gel transition material (Jiang et al., 2023). Gelatin-based temperature-responsive drug delivery vehicles have been developed as an adjunct to cancer treatment (Nardecchia et al., 2019). Gelatin is water-soluble because of several hydrophilic groups on the surface to form many hydrogen bonds (Mikhailov, 2023; Yildirim et al., 2023). It is more widely used than other biological materials because of its diverse source composition and low immunogenicity (Yang et al., 2022). Medical hemostatic gelatin—purified gelatin extracted first from animal skin in 1945—possesses a porous structure capable of absorbing 45 times heavier than its own weight; its porous structure absorbs blood and expands, destroys platelets, promotes the formation of blood clots, and seals the blood vessel crack or wound by forming a coagulation grid to achieve hemostasis (Huang et al., 2020). With the development of nanomedicine, gelatin is crucial in anti-cancer drug delivery, wound dressings, food safety, bone regeneration, and tissue engineering (El-Seedi et al., 2023; Ju et al., 2023; Tan et al., 2023).

ECM serves as a dynamic environment for cell survival and is crucial in cell growth, mechanical support, and nutrient supply (Guvatova et al., 2022; Mohindra et al., 2022). Besides supporting parenchymal cells, ECM is vital in cell proliferation, differentiation, and migration. Structural and functional changes in the skin ECM often lead to severe skin diseases, most commonly skin fibrosis (Wang et al., 2023). ECM comprises collagen, glycosaminoglycans, elastin, heparan sulfate, chondroitin sulfate, and water (Malta et al., 2022). As previously described, gelatin is a breakdown product of natural collagen (Tan et al., 2018). Gelatin compensates for tissue breakage around the wound and provides a natural substrate for ECM. Therefore, gelatin is an ideal material to promote skin healing.

## 4 Gelatin-based biomaterials promote wound healing

### 4.1 Hemostasis

Bleeding is a direct manifestation of skin damage, and uncontrolled bleeding is the leading cause of trauma death (Huang et al., 2022b). Timely and effective hemostasis is a prerequisite for wound healing. Gelatin has adhesive properties that facilitate facilitate wound healing. Conventional dressing applications are severely limited by uncontrolled gel times and low mechanical properties (Tu et al., 2019). Liang et al., 2022 developed a gel adhesive with gelatin, sodium alginate (SA), and protocatechualdehyde. The adhesion properties are brought by the strong interaction between the amino and carboxyl groups of gelatin and SA (Tarakhovskaya, 2014). The gel adhesive had an energy storage modulus of 2400 Pa, making it a self-healing material because of the Schiff base reaction between the aldehyde/quinone and amino groups and the strong hydrogen bond between gelatin and SA. *In vivo* experiments in a mouse hepatocellular carcinoma hemorrhage model, the gel binder had less bleeding and shorter hemostasis than the control group. In an experiment on skin wound healing in mice, the gel binder promoted complete wound healing at 21 days.

**FIGURE 1:**
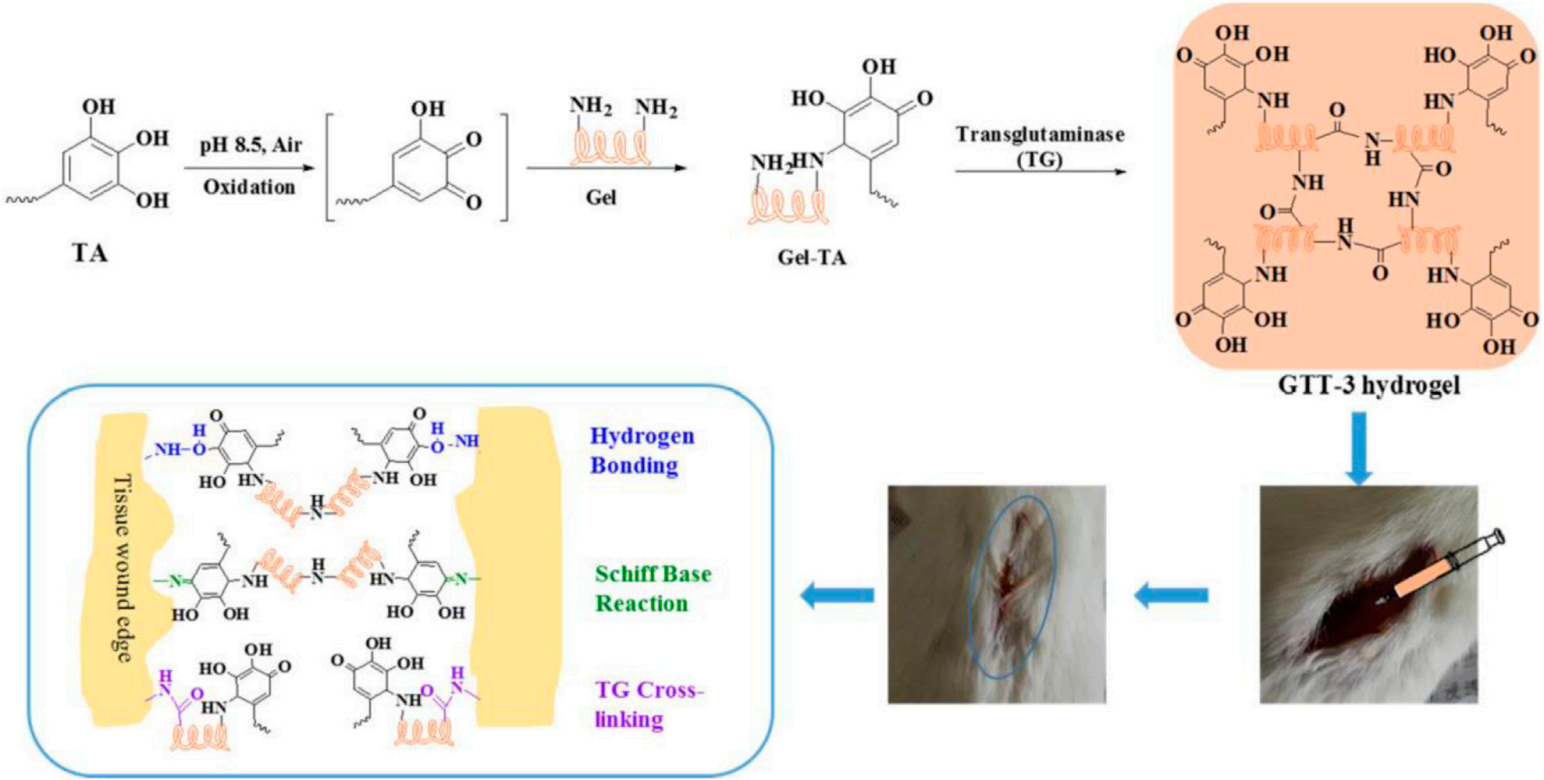
Diagrammatic Sketch of Enzyme-Induced Cross-Linking Hydrogel with the Ability of Wound Adhesion and Post-Traumatic Healing Protection. Reproduced with permission from (Zhou et al., 2021).

For wound hemostasis, adsorption is a crucial property of hemostatic materials (Yang et al., 2017). When the adsorption rate is low or slow, it is difficult to achieve rapid hemostasis of the wound (Zhang et al., 2018a). To enhance the adsorption of gelatin, Wang et al. mixed chitosan with gelatin and crosslinked with proanthocyanidins (PC) to make chitosan/gelatin sponge (PCGS) (Sun et al., 2023). PC—a naturally occurring polyphenol—is widely used in biomedicine because of its high antioxidant and antibacterial properties (Wang et al., 2019). PC addition to PCGS enlarge its porous morphology, thereby increasing its adsorption. PCGS had a lower hemolysis rate than chitosan because PC reduces the cation level on the surface of chitosan in PCGS, thereby reducing the interference with coagulation factors. An *in vivo* experiment confirmed that a PCGS group effectively reduced bleeding and promoted wound healing on day 14. Tannins (TA) are natural plant polyphenolic compounds that bind to proteins through hydrophobic action and hydrogen bonding, thereby exhibiting strong adhesion, antioxidant, antibacterial, and procoagulant properties (Ninan et al., 2016). Zhou et al. crosslinked TA-modified gelatin with glutaminyltransferase (TG) to produce crosslinked hydrogels (Figure 1) (Zhou et al., 2021). TG addition can enhance the covalent binding with amino acids in gelatin and increase the stability of the cross-linked hydrogels. The cross-linked hydrogels had a slight swelling ratio (556.8%), but they did not cause compression to surrounding tissues. The solubility of the cross-linked hydrogels was 78.8%, providing growth space for cell proliferation. The cross-linked hydrogels can continuously release TA around the wound, which can cause platelet aggregation and activation of coagulation factors. In a rat tail vein hemostasis model, the mean hemostasis time of the cross-linked hydrogels (241 ± 52.7 s) was lower than a control group (323.48 ± 82 s). Dopamine (DA) has a hydrophore and amine group and can form hydrogen bonds with substrates to induce adsorption (Chou et al., 2021). DA/gelatin can enhance the adsorption of gelatin-based hydrogels, thereby enhancing their water absorption, which is conducive to wound hemostasis. Luo et al., 2022 DA/gelatin and hyaluronic acid (HA) to produce hemostatic hydrogels. HA is a natural mucopolysaccharide, but its weak mechanical properties and rapid degradation characteristics limit its clinical application (Bao et al., 2021). Compared with gelatin hydrogels, hemostatic hydrogels have higher mechanical strength and can maintain their shape for 7 days in water. Hemostatic hydrogels have a highly cross-linked nano-microporous structure to provide a cross-linked microenvironment. Hemostatic hydrogels have good biocompatibility and is beneficial to cell adhesion and proliferation. The adhesion strength of the hemostatic hydrogels was 137 ± 16 kPa. In an internal bleeding model, the hemostatic hydrogels achieved rapid hemostasis within 135 s.

Huang et al., 2022c lyophilized gelatin soaked in a solution of silver nanoparticles (Ag NPs) to construct a degradable GT/Ag freeze gel, which had a compressive strength of 7 kPa. GT/Ag gels have good mechanical properties and can stably release Ag ions to achieve their antibacterial effect. GT/Ag gels have a large swelling rate of more than 4,000% and can adsorb a large amount of exudate around the wound. AgNPs have a particle size of 10–20 nm, which can enhance the porosity of GT/Ag gels in gelatin, enhance the gas exchange ability around the wound, and promote wound healing. In mouse models of hepatic hemorrhage, the rapid blood absorption and local hemoconcentration of GT/Ag gels promoted platelet and blood cell aggregation. Furthermore, AgNPs can promote the antibacterial effect around the wound and accelerate wound healing. ε-Poly-l-lysine (EPL) has a rapid hemostatic effect by electrostatically adsorbing red blood cells and activating platelet aggregation (Tang et al., 2020). However, the poor biocompatibility of EPL limits its clinical application (Li et al., 2017). Zhang et al. crosslinked oxidized dextran (ODex) with EPL and gelatin methacryloyl (GelMA) via the Schiff base reaction and further crosslinked GelMA using UV irradiation to prepare double-network (DN) hydrogels (Zhang et al., 2022). The porosity and swelling rate of the DN hydrogels were above 70% and 400%, respectively, and the DN hydrogels promoted cell proliferation and migration and the absorption of wound exudate. The DN hydrogels had extremely high tensile strength (16.9 ± 0.44 kPa) and good elastic retraction. In a rat hepatic hemorrhage model, the DN hydrogels stopped bleeding *in situ* at 38.3 ± 4.1 s. Subsequently, under UV irradiation, the DN hydrogels underwent rapid gelation combined with an adhesion effect, producing a good sealing effect at the bleeding site and achieving hemostasis.

### 4.2 Antibacterial properties

Multidrug-resistant bacteria (*Staphylococcus aureus* and *Escherichia coli*) proliferate rapidly at the wound site and secrete many extracellular polymeric substances (EPS) to form biofilms to effectively prevent antibiotic penetration and resist host immunity, exacerbating wound infection and non-healing (Liang et al., 2019). The antimicrobial ability of gelatin alone is weak, and its mechanical properties are poor; therefore, there is an urgent need for improved gelatin-based biomaterials to improve the antimicrobial properties around the wound (Ndlovu et al., 2021). Tetracycline hydrochloride (TH) is a spectrum antibiotic commonly used in clinical practice, exhibiting a strong bacteriostatic effect on various bacteria (Chen et al., 2020). Curcumin (Cur) promotes cell proliferation, migration, and collagen deposition and can also reduce ROS (reactive oxygen species) around the wound to achieve anti-inflammatory effects (Rezaii et al., 2019). However, Cur often limits its clinical use because of its low bioavailability (Wibowo et al., 2020). Xia. et al., 2022c loaded Cur into a polycaprolactone (PCL) core and TH into a gelatin shell to prepare a PCL-Cur/GEL-TH core-shell nanofiber membrane. Compensating for PCL’s slow degradation, gelatin addition could increase the biodegradability and biocompatibility of the PCL-Cur/GEL-TH core-shell nanofiber membrane. The shell structure of the PCL-Cur/GEL-TH membrane releases TA for antibacterial action, and Cur released in the inner core has an anti-inflammatory effect. The PCL-Cur/GEL-TH membrane has good biocompatibility and does not affect surrounding normal cells. The PCL-Cur/GEL-TH membrane had a diameter of 178 ± 25 nm, tensile strength of 6.43 ± 0.14 MPa, and Young’s modulus of 12.77 ± 0.02 Mpa. It released 29.87% and 88.79% of Cur and TH, respectively, within the first 2 h to meet the early antibacterial requirements around the wound.

**FIGURE 2:**
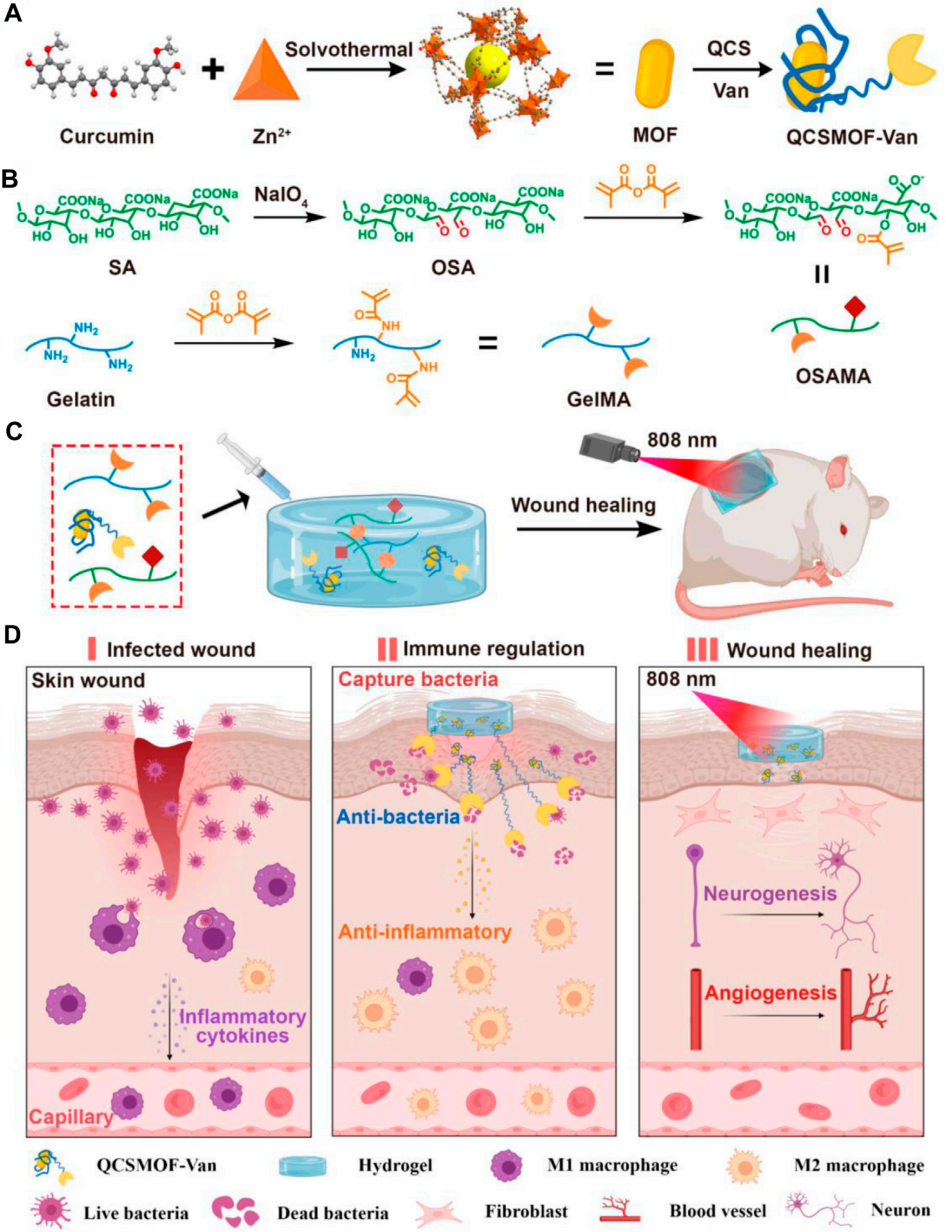
Schematic Illustrations of **(A)** Synthesis of QCSMOF-Van, **(B)** Synthesis of GelMA and OSAMA, **(C)** Synthesis of QCSMOF-Van Hydrogel for Repairing Chronic Wounds, and **(D)** Mechanism of QCSMOF-Van Hydrogel for Repairing Chronic Wounds. Reproduced with permission from (Huang et al., 2022d).

Metal-organic frameworks (MOFs) have rapidly developed in the biomedical field with large specific surface areas and high porosity (Wang et al., 2018b). As a commonly used metal ion in MOFs, zinc ions can promote cell growth, differentiation, hair follicle growth, and nerve regeneration (Gupta et al., 2016). Zinc ions can also promote macrophage polarization to achieve antibacterial effects (Porfire et al., 2014). Huang et al. used Cur-containing MOFs to load vancomycin (Van) and coated the skeleton with quaternary ammonium salt chitosan (QCS) to produce QCSMOF-Van (Figure 2) (Huang et al., 2022d). Methacrylic anhydride-modified gelatin can be achieved by trapping bacteria with a positive charge on the surface of the QCS, resulting in the antimicrobial effect of TI’gao QCSMOF-Van. QCSMOF-Van has good biocompatibility and degradability. The QCS on the surface of QCSMOF-Van contains a positive charge, which can catch bacteria with a negative charge, and under the combined action of zinc ions and Aan, QCSMOF-Van can achieve excellent antibacterial properties. Furthermore, QCSMOF-Van had high adhesion strength (34.146 ± 5.032 kPa) and compressive strength (74.667 ± 9.504 kPa). An *in vitro* experiment confirmed that QCSMOF-Van killed *S. aureus* (antibacterial rate, 99.953% ± 0.080%) and *E. coli* (antibacterial rate, 98.676% ± 0.654%) within 24 h. An *in vivo* experiment to promote wound healing confirmed that QCSMOF-Van promoted complete wound healing within 21 days. Zhang et al. used an iron-containing metal-organic framework (Fe-MOF) to release Gox (glucose oxidase) in a controlled manner by coupling Gox through amide bonding and using PCL, gelatin, and d-glucose (PGD) fiber networks to produce G@Fe/PGD fiber membranes (Zhang et al., 2023). The GOx released by the G@Fe/PGD fiber membranes oxidizes glucose to gluconic acid and hydrogen peroxide, which can decompose hydrogen peroxide and produce radical dot-OH amount under the action of Fe-MOF, thereby improving the antibacterial effect of the G@Fe/PGD fiber membranes. Gelatin addition can increase the biodegradability of G@Fe/PGD fiber membranes. In a rat model of infected wounds, G@Fe/PGD fiber membranes achieved complete wound healing within 14 days. Huang et al. developed injectable adhesive hydrogels using polyoxometalates (AgPOM), urea, gelatin, and tea polyphenols (TPs) (Huang et al., 2023). Urea is used as a regulator to control the cross-linking of gelatin and TPs and can penetrate into tissues to form strong adhesion and prevent bacterial invasion. An *in vitro* experiment demonstrated that the release of AgPOM from adhesive hydrogels killed 90% of bacteria and effectively promoted wound healing within 14 days.

Fucoidan (Fuc) has good biocompatibility and exhibits good antibacterial, antioxidant, and immunomodulatory functions because of its rich sulfuric acid group (Huang et al., 2022d). Lu et al., 2022a mixed TA into a mixture of gelatin and Fuc to make Gel&Fuc-TA hydrogel wound dressing. Gel&Fuc is connected to TA by hydrogen bonding, and after dialysis and lyophilization, the Gel&Fuc-TA hydrogels had a porous structure (1.9–5.26 μm). Gel and Fuc-TA continuously release TA and Fuc in the body without causing hemolysis. Compared with a gelatin-alone group, the Gel&Fuc-TA hydrogels killed >95% of *E. coli* and *S. aureus* in 4 days. To enhance the adhesion of the hydrogel dressing, Li et al. (2023a) used a mixture of polyacrylamide (PAAm) and poly (N-isopropyl acrylamide) (PNIPAm) to form mucus for the delivery of Ag/gelatin-TA NPs to form DN hydrogels. The DN hydrogels have excellent adhesion and biocompatibility because of their micro-chuck structure. Due to the photothermal effect of Ag-Ta NPs and the gel-sol transition of gelatin, DN hydrogels release Ag and TA and have excellent antimicrobial properties. Chen et al., 2023a used 3D printing technology to produce hollow channel hydrogel scaffolds from a mixture of DA-modified alginate (Alg-DA) and gelatin. Copper and calcium ions are evenly distributed within the hydrogel holder. The compressive strength of the hydrogel stent was 1.42 ± 0.24 MPa. Copper ions can promote vascular regeneration; after crosslinking copper ions and calcium ions, hydrogel scaffolds show good photothermal effects. The higher the copper ion, the stronger the photoconversion effect. The hydrogel scaffolds killed 95% of *S. aureus* and *E. coli* around the wound, indicating their effective antibacterial activity against gram-positive and gram-negative bacteria (Dai et al., 2021).

### 4.3 Anti-inflammatory properties

Cell proliferation around the wound produces higher reactive oxygen species (ROS) and lower pH (Deng et al., 2018). Large bacterial colonization of wounds and endotoxin production can lead to inflammatory factors (Chin et al., 2019). Inflammatory factors can recruit inflammatory cells to migrate around the wound to engulf bacteria and necrotic material (Akita, 2019). Proper inflammation benefits wound repair, but too many inflammatory cells can invade normal tissue. Nimesulide (NIM) is a widely used nonsteroidal anti-inflammatory drug (Zheng et al., 2000). Phenylboronic acid (BA) derivatives can be targeted in response to ROS by reversible covalent bonds. Wang et al., 2021 grafted 3-carboxyphenylboronic acid on the gelatin molecular framework, embedded NIM in hydrogels with polyvinyl acetate as a crosslinker and successfully synthesized drug-loaded hydrogels with an inflammatory response. Under high ROS of inflammation, drug-loaded hydrogels can release NIM on demand to inhibit inflammatory response and promote wound healing. Lu et al., 2022b used trivalent iron (Fe^3+)^ and 2, 3, 4-trihydroxybenzaldehyde (THA) to form a complex (THA&Fe), which was then linked with tilapia skin gelatin (Tsg) through Schiff base to form Tsg-THA&Fe gelatin hydrogels. Gelatin addition accelerates gelation by Schiff base. The degradable Tsg-THA&Fe completely degraded in 16 days in the body. Studies have confirmed that Tsg-THA&Fe has good biocompatibility and did not cause a significant hemolytic reaction. Tsg-THA&Fe has good free radical scavenging capacity (>80%) and antioxidant activity. Tsg-THA&Fe promotes the polarization of macrophages from M1 to M2 and reduces the expression of tumor necrosis factor-α (TNF-α), interleukin-6 (IL-6), interleukin-1β (IL-1β), and interleukin-10 (IL-10). Chen et al. fabricated hybrid hydrogels of GelMA and PDA-coated fullerene (C60@PDA) for wound repair (Chen et al., 2023b). The C60@PDA hydrogels had good biocompatibility and were degraded entirely *in vivo* in 9 days. The C60@PDA hydrogels scavenged free radicals and removed 96% of ROS around the wound within 77 h. The C60@PDA hydrogels reduced the *in vivo* expression of IL-6, TNF-α, VEGF, and TGF-β for 6 days.

Bioactive glass (BG) is a biomaterial with biological activity (Zhang et al., 2018b). As a type of BG, 45S5 releases Ca, Si, and P ions to promote wound healing (Yu et al., 2016). Tomar et al. developed multifunctional composite hydrogels using gelatin, chitosan, diclofenac-loaded BG, PDA-coated BG, and Cur-AgNPs (Tomar et al., 2023). Gelatin, chitosan, and PDA coatings form stable and porous scaffolds that form the basis for BG attachment. The BG hydrogels had a size of 300–400 nm and a charge of −27.4 mV, with a strong specific surface area and drug loading efficiency. The BG hydrogels continuously released 76% diclofenac sodium within 168 h, thereby achieving anti-inflammatory effects on wounds. Moreover, the multifunctional complex hydrogels released 58% of Cur-AgNPs in the body for 58 h to achieve the antibacterial effect. The multifunctional complex hydrogels have good biocompatibility, promote cell proliferation around the wound, and do not cause adverse reactions to the wound. In mice, the multifunctional complex hydrogels reduced inflammatory cell infiltration and the expression of inflammatory factors. Compared with a gelatin-alone group, the multifunctional complex hydrogels had the advantages of inducing angiogenesis, regulating inflammation, eliminating microorganisms, and relieving pain. Ren et al. grafted phenylboronic acid (BA-HA) with polyvinyl acetate (PVA) and HA to form PH/GMs@bFGF&PDA by encapsulating PDA and gelatin microspheres containing basic fibroblast growth factor (GMs@bFGF) (Ren et al., 2023). Gelatin addition provides the PH/GMs@bFGF&PDA a strong adhesion (101.46 ± 10.88 kPa), which keeps the hydrogels adhering to the wound. The PH/GMs@bFGF&PDA complex formed a porous gel structure with a pore size of 5–30 μm within 120 ± 7 s of injection around the wound. The long-empty structure of the PH/GMs@bFGF&PDA complex provides attachment points for PDAs and bFGF. The PH/GMs@bFGF&PDA complex continuously releases bFGF in the body, and the release rate of bFGF reached 67.5% ± 21.7% within 69 days and reduced the infiltration of inflammatory cells within 21 days. Moreover, the release of bFGF increases collagen production and deposition, promotes angiogenesis, and accelerates wound healing.

### 4.4 Promotion of vascular regeneration

Neoangiogenesis is crucial from the initial wound healing to the wound remodeling phase (Wilkinson and Hardman, 2020). New blood vessels provide oxygen for the organic granulation tissue around the wound. Hypoxia causes sustained activation of peri-wound hypoxia-inducible factor 1 (HIF1), which promotes angiogenic factor (VEGF) (Hong et al., 2014). HIF1 can increase ROS production, persistent inflammation and delaying wound healing. ROS also degrades collagen (Chen et al., 2012). Fat-derived mesenchymal stem cells (ADSCs) can produce exosomes paracrinely, promoting angiogenesis and chronic wound healing (He et al., 2020). Li et al., 2023b mixed hypoxic-conditioned ADSCs-conditioned medium (cADSC-CM) with GelMA to produce GelMA + cADSC-CM hydrogels. GelMA increases the loading rate of stem cells because of its injectable and UV cross-linking properties. The pore size of the GelMA + cADSC-CM hydrogels was 342.3 μm. On day 4 of an *in vivo* experiment, hypoxic-conditioned ADSC promoted angiogenesis by activating HIF1α secretion of VEGF. Studies have shown that GelMA + cADSC-CM hydrogels can increase the number of microvessels compared with a blank control group.

Borate bioactive glass (BBG) promotes angiogenesis and promotes wound healing (Balasubramanian et al., 2017). Alg is hydrophilic, biocompatible, and wound exudate absorbent (Biswal et al., 2016). However, the poor cell adhesion of Alg limits its clinical application. Monavari et al. partially oxidized Alg to obtain alginate dialdehyde (ADA) and used 3D printing technology to combine ADA with gelatin and incorporate BBG to obtain ADA-GEL/BBG (Monavari et al., 2023). ADA mixed with gelatin enhances cell adhesion. The ADA-GEL holder has a porous structure with BBG particles as ink, and its size ranges from 10 to 100 μm. Compared with ADA-GEL (8 kPa), BBG addition significantly increased the compressive strength of the ADA-GEL/BBG hydrogel (14 kPa). However, the ADA-GEL/BBG hydrogel reached 25% degradation after immersion in solution for 14 days, indicating that the hydrogel has good biocompatibility with degradation. The BBG released by ADA-GEL/BBG not only has a rough surface, providing an attachment point for cell proliferation but also stimulates the production of VEGF and promotes vascular regeneration.

Liu et al., 2023b developed multifunctional bilayer microneedles (DMN@TH/rh-EGF) using gelatin carboxymethyl chitosan (Gel-CMC) loaded with TH and recombinant human epidermal growth factor (rh-EGF). rh-EGF promotes angiogenesis, collagen deposition, and tissue regeneration (Chi et al., 2020). With a height of the DMN@TH/rh-EGF microneedles of ≈780 μm, DMN@TH/rh-EGF enabled rapid release of TH and rh-EGF with a cumulative release percentage of up to 5.20% at 91 min. The released TH reduces inflammation around the wound and kills bacteria, and rh-EGF promotes cell migration and capillary network formation. Compared with a control group without blank, the DMN@TH/rh-EGF microneedles formed abundant blood vessels within 14 days, supporting oxygen and nutrient transport. Zhao et al., 2023 produced CvMN from polyvinyl acetate (PVA) and GelMA encapsulated with *Chlorella vulgaris* (Cv) to promote diabetic wound healing. Cv has good biocompatibility, can be broken down to produce oxygen, and can regulate immunity and antioxidants (Lee et al., 2019). CvMN can be released around the wound, relieve the lack of oxygen around the wound, and promote the regeneration of capillaries around the wound.

Hydrogen sulfide (H_2_S) can promote wound angiogenesis and improve wound healing environment (Akter, 2016). However, the gaseous arrest and short half-life of H_2_S limit its clinical application (Hu et al., 2016). Xiao et al. combined keratin with H_2_S to produce H_2_S donors by thiol-disulfide exchange reaction and combined polyurethane (PU) and gelatin using electrospinning technology to prepare composite pads for carrying H_2_S (Han et al., 2022). Gelatin addition enhances the biocompatibility and biodegradability of the composite. The composite pad is highly absorbent, absorbing 500% of its volume of water while continuously releasing H_2_S. An *in vivo* experiment findings suggest that the composite pads can accelerate the formation of granulation tissue, enhance collagen deposition, and promote angiogenesis, thereby improving wound healing. Xie et al. developed PLGA/gelatin fiber membrane scaffolds (FMS) using electrospinning technology to deliver mesenchymal stem cells loaded with adipose (Hsieh et al., 2022). Gelatin addition can increase the biodegradability and biocompatibility of FMS. Stem cells can promote the production of transforming growth factor-β3 (TGF-β3), VEGF, and insulin growth factor (IGF) through paracrine, promoting cell migration and vascular regeneration (Chen et al., 2021). Yu et al., 2023 reduced graphene oxide (GOG) by gelatin to increase its compressive strength for the delivery of N-acetylcysteine (NAC) to obtain NAC-GO-Gel. GO-Gel stents have good biocompatibility and promote neovascularization (Shen et al., 2022). NAC is a reduced glutathione (GSH) precursor that promotes wound healing in the body (Lasram et al., 2015). The NAC-GO-Gel hydrogels had an average particle size of 753 ± 80 nm and Young’s modulus of 94.13 ± 3.25 MPa. On day 14 *in vitro*, the NAC-GO-Gel hydrogels released NAC (68.68% ± 6.33%) to promote wound angiogenesis, reduce inflammatory response, and promote wound healing.

### 4.5 Promotion of epidermal regeneration

Astragalus polysaccharides (APS) have anti-inflammatory and vascular regeneration effects (Liu et al., 2011). Wen et al. made nanofiber membranes (GEL/APS NFM) using electrospinning technology with gelatin and APS for wound healing (Wen et al., 2023). Gelation addition gives the nanofiber membrane satisfactory stretchability and ideal wound healing efficiency. NFM has a similar structure to ECM and has strong tensile strength, which is widely used in wound dressings. The average diameter of GEL/APS NFM was 773 nm, and its elongation reached 514.6% as the hydrogen bond formed between gelatin and APS increased the tensile resistance of GEL/APS NFM. GEL/APS NFM had good biocompatibility, and the cell viability was more than 90% with cell culture for 72 h. Compared with a blank control group, GEL/APS NFM *in vivo* for 15 days increased the expression of VEGF and promoted vascular epithelial regeneration. Moreover, GEL/APS NFM promotes collagen deposition and epidermal regeneration. Li et al., 2022 used GelMA to mount umbilical cord mesenchymal stem cells (UC-MSCs) to make gelatin microspheres. The UC-MSCs released by gelatin microspheres can promote the release of basic fibroblast growth factor (bFGF), fibroblast adhesion, proliferation, ECM remodeling, and collagen deposition.

Epidermal growth factor (EGF) is crucial in skin metabolism, and the lack of EGF around the wound can lead to wound non-healing (Chen et al., 2019). Yang et al., 2023 used GelMA to deliver zinc and magnesium particles to produce GelMA/Mg/Zn. Gelatin addition enhances the biocompatibility and biodegradability of the composite. Zinc ions promote epithelial regeneration and accelerate collagen deposition (Lin et al., 2018). Magnesium promotes angiogenesis and accelerates fibroblast proliferation and adhesion (Amberg et al., 2018). GelMA/Mg/Zn has a porous structure with a pore size of 20–100 μm, and Zn and Mg can be uniformly mixed into the composite. GelMA/Mg/Zn has good biocompatibility and degradability. With the degradation of GelMA/mg/Zn, Zn and Mg can be released around the wound. Compared with a control group, the GelMA/Mg/Zn hydrogels showed new collagen fibers at the wound site on day 7, all newly generated epithelial tissue. The GelMA/Mg/Zn hydrogels *in vivo* for 14 days promoted the increase of wound EGF and wound healing, and the wound healing rate was 98.9% ± 1.4%. Silk fibroin (SF) is a high protein crystallization provided by fairly large hydrogen bonds and β sheet crystals, which has good elasticity (Kim et al., 2020). Xu et al., 2023a used 3D printing technology to combine SF and GelMA into G-S hydrogels. The G-S hydrogels had a porous structure with average pore sizes of 226.54 ± 41.86 μm. G-S hydrogels have good biocompatibility and can be degraded *in vivo*. The G-S hydrogels *in vivo* for 14 days promoted collagen deposition, cell proliferation, and epidermal reepithelialization compared with a blank control group, and the epidermal thickness increased by approximately 147.94 ± 17.37 μm.

## 5 Clinical application of gelatin materials to promote wound healing

Absorbable gelatin hemostatic sponges are currently used in clinical settings (Hajosch et al., 2010; Ehab et al., 2020). Gelatin used in clinical practice mainly relies on its hemostatic effect and is widely used in intraoperative and postoperative patients. Gelatin sponge has good histocompatibility and resorbability. It is a loose, porous sponge-like material with strong water absorption ability; its porous structure can absorb blood expansion, destroy platelets, release coagulation-activating enzymes, accelerate coagulation, promote the formation of blood clots, and achieve hemostasis (Signorelli and Montano, 2020). Jin et al. explored absorbable gelatin sponges (Cutanplast; Mascia Brunelli S.p.A., Milan, Italy), which have rapid hemostasis and do not have adverse effects (Hajosch et al., 2010). Bahar Alkaya et al., 2023 demonstrated the ability of absorbable hemostatic gelatin sponge (Spongostan^®^, Clinisponge, Turkey) to promote wound healing in gingival graft patients. Furthermore, studies have demonstrated the hemostatic effects of hemostatic gelatin such as Surgicel^®^ (Johnson & Johnson, New Brunswick, NJ), Evicel Fibrin Sealant^®^ (Ethicon, Somerville, NJ), and FloSeal^®^ (Baxter) (Signorelli and Montano, 2020).

Despite being from different production companies, the effectiveness of gelatin hemostatic sponges has been recognized by clinicians. Combined application of gelatin materials has a stronger hemostatic effect than gelatin alone (Femminella et al., 2016). Gelatin-based material combination therapy has been reported. Naoki Morimoto et al., 2015 treated chronic skin ulcers with a combination of gelatin and platelet-rich plasma (PRP). PRP contains a large amount of platelet-derived growth factor (PDGF), transforming growth factor β (TGF-β), and vascular endothelial growth factor (VEGF), which can accelerate chronic wound healing (Xu et al., 2023b). Jason et al. developed a hemostatic agent based on the formation of gelatin *in situ* gel, in which the amino acid sequence is cross-linked by a novel microbial transglutaminase distinct from commercial mTGase (Wright et al., 2014). The hemostatic agent is beneficial because its cascade reaction with coagulation produces a biochemical cross-linking effect, which has stronger adhesion, elasticity, and hemostatic effect than SURGIFLO^®^ (Wright et al., 2014).

## 6 Conclusion and outlook

Gelatin-based wound healing dressings are evolving rapidly because of their biocompatibility and clear advantages in delivery. The delivery efficiency of gelatin carriers is highly influenced by gelatin type, concentration, and formulation conditions. Preclinical studies have widely demonstrated that gelatin serves as a drug delivery carrier while maintaining its original activity. Gelatin-based electrospinning fibers, hydrogels, sponges, stents, films, and other technologies make it highly absorbent and highly drug-release efficient around the wound. Gelatin has advantages in promoting hemostasis, anti-infection, anti-inflammatory properties, vascular regeneration, and epithelial tissue regeneration. It has made great progress in promoting chronic wound healing. However, wound healing is a chronic and changing complex environment primarily associated with appropriate compression, exudative management, pressure, and tension to control chronic wounds. Although gelatin-based biomaterials can solve some difficulties, they are substantial challenges. There has been considerable interest in using 3D printing technology to prepare gelatin scaffolds to promote wound healing. 3D-printed gelatin can provide good mechanical and compressive strength around the wound and deliver biological factors (VEGF and bFGF) to promote chronic wound healing. Medical gelatin hemostatic materials have been applied in clinical treatment because they are irreplaceable in hemostasis. There are many reports on gelatin hemostatic materials produced by listed companies. It was only in 1984 that the US FDA approved the first commercial fibrin glue (Tisseel). However, there have not been many reports on the anti-inflammatory properties of gelatin, promoting angiogenesis. However, the use of gelatin for wound treatment remains at an early stage, and large-scale clinical trials to verify its effectiveness are lacking. However, although the hemostatic effect of synthetic gelatin hemostatic materials has been proven, the complex processing process also increases its selling price. Future studies should focus on improving the efficiency of gelatin-based biomaterials for wound healing. We believe that the widespread application of gelatin-based biomaterials is just around the corner.
